# Design and implementation of PLA/GO/metal oxide composites for CO_2_ sensing application

**DOI:** 10.1038/s41598-025-89337-x

**Published:** 2025-02-17

**Authors:** Khaled S. Amin, Mohamed M. Yassin, Yahia M. Abdallah, Yusuf M. Alsayyad, Ahmed F. Mabied, Hanan Elhaes, Medhat A. Ibrahim

**Affiliations:** 1 Physics Department, Faculty of Science, Al-Azhar University, Cairo, Egypt; 2 X-ray Crystallography lab, Solid State Physics Department, National Research Center, 33 Bohouth Street, Dokki, 12622 Cairo Egypt; 3 Physics Department, Faculty of Women for Arts, Science and Education, Ain Shams University, Cairo, 11757 Egypt; 4 Spectroscopy Department, National Research Centre, 33 El-Bohouth St., Dokki, 12622 Giza Egypt; 5 Molecular Modeling and Spectroscopy Laboratory, Centre of Excellence for Advanced Science, National Research Centre, 33 El-Bohouth St., Dokki, 12622 Giza Egypt

**Keywords:** PLA, GO, Gas sensor, CO_2_ sensor, Nanocomposite, Materials science, Physics

## Abstract

This study investigates the modification of polylactic acid (PLA) by the incorporation of graphene oxide (GO) and metal oxides (ZnO and CuO), with the aim of developing efficient CO₂ sensors. Key properties, including total dipole moment (TDM), energy gap (ΔE), molecular electrostatic potential (MESP), and density of states (DOS), were calculated using density functional theory (DFT) to gain insight into the interactions between the composites and CO₂ gas. Experimental techniques such as X-ray diffraction (XRD), Fourier transform infrared spectroscopy (FTIR), and optical confocal microscopy were used to validate the material composition and bonding mechanisms. The analysis revealed the presence of SiO₂ impurities in the PLA matrix, which could potentially affect the sensing behavior of the composite. The composites demonstrated effective CO₂ sensing capabilities in experimental tests. This combined theoretical and experimental approach demonstrates that PLA/GO/metal oxide composites offer significant potential for sustainable CO₂ sensing, contributing to air quality monitoring and greenhouse gas regulation.

## Introduction

Polylactic acid (PLA) is a biopolymer derived from renewable sources, including corn, which has become a promising material for a variety of applications in both engineering and the medical field^1^. PLA is renowned for its eco-friendly credentials and its capacity to supplant conventional polymers derived from petrochemicals^2^. A notable benefit of PLA is its biodegradability, which occurs primarily through hydrolytic degradation. This process involves the breakdown of ester bonds affected by moisture, light, temperature, and microorganisms. Furthermore, exposure to ultraviolet radiation contributes to photodegradation. To enhance PLA’s resistance to environmental degradation while maintaining its biodegradability, the incorporation of natural stabilizers is a viable strategy^3^.

Another noteworthy attribute of PLA is its minimal energy consumption during the manufacturing process, which makes it a cost-effective and environmentally sustainable option for production. As a material derived from renewable resources, PLA provides a sustainable alternative that also facilitates the sequestration of CO₂ emissions, thereby contributing to the reduction of greenhouse gases^4^. These properties align particularly well with industrial applications of CO₂ sensors, where sustainability and environmental impact are growing concerns. For example, incorporating PLA-based composites into industrial CO₂ monitoring systems can reduce the overall carbon footprint associated with sensor production and disposal, aligning with circular economy principles. Furthermore, the physical and mechanical properties of PLA can be modified through a variety of techniques, thereby enabling its use in a diverse array of applications. This versatility renders PLA a valuable material in the pursuit of more sustainable and eco-friendly products^5^ combining this essential polymer with other advanced materials has the potential to enhance the aforementioned properties and resolve the identified limitations.

Graphene oxide (GO), the oxidized form of graphene, is distinguished by a high surface area and exceptional thermal stability^6,7^. The surface of GO has been functionalized with oxygen-based functional groups, including hydroxyl (-OH), alkoxy (C-O-C), carbonyl (C = O), and carboxyl (-COOH), which impart a variety of functional properties^8^. The distinctive physical, chemical, and mechanical properties of GO are a consequence of its functionalization, rendering it a promising material for a multitude of applications. The presence of the COOH group endows GO with unique characteristics that make it a valuable addition to a diverse range of technological and industrial processes^9–11^.

Polymer-GO nanocomposites, produced through various methods, offer a multitude of applications in diverse fields, including filtration, sensors, engineering scaffolds, wound healing, and tissue engineering^12^. The remarkable properties of graphene can be transferred to its composites with polymers. The PLA/GO nanocomposite displays notable improvements in mechanical strength, thermal stability, gas barrier properties^13^, electrical conductivity^14^, and flame retardancy relative to the original polymer. Graphene oxide (GO) nanocomposites have the potential to facilitate the development of advanced polymeric materials with enhanced properties. By optimizing the interaction between graphene oxide (GO) and the polymer matrix^15^, metal oxide-based composites have demonstrated potential as gas sensors for a range of gases, including CO₂^16,17^. For instance, research has explored CuO-based composites for CO₂ sensing, achieving good sensitivity at low temperatures^18^. Nevertheless, there is a sustained endeavor to develop novel materials with enhanced performance characteristics^19^.

Given its pivotal role in climate change, CO₂ sensing is of paramount importance for the monitoring and regulation of greenhouse gas emissions. Moreover, CO₂ sensors have a multitude of applications that can enhance air quality control in buildings and optimize industrial processes. It is of paramount importance to develop novel and efficient CO₂ sensors with enhanced performance in order to effectively address these challenges^20^.

In our previous research, we employed the density functional theory (DFT) method to examine the properties of PLA/GO/ZnO and PLA/GO/Cu_2_O composites as gas sensors. Our investigation encompassed a range of gases and yielded promising outcomes, as documented in our previous publication^21^. Building upon the aforementioned foundation, this study explores the potential application of our composites as CO_2_ sensors using a combined computational and experimental approaches. An analysis of the commercially available PLA revealed the presence of SiO₂ as an impurity. The introduction of SiO₂ during the manufacturing process may influence the properties of the PLA-based composite. Copper oxide (II) CuO is employed in place of Copper oxide (I) Cu_2_O due to its availability during the experimental phase. While Cu₂O was used in our previous theoretical study, CuO shares similar semiconducting properties and has been widely reported as an effective material for gas sensing^22,23^ and, particularly for CO₂^24^. The theoretical section will concentrate on calculating the total dipole moment (TDM), band gap energy (ΔE), and molecular electrostatic Potential (MESP) in addition to mapping the density of states (DOS). In order to confirm the presence of the material and to verify the bonds, as well as to elucidate the interaction mechanism, X-ray diffraction (XRD) will be utilized in an experimental context. Fourier-transform infrared spectroscopy (FTIR) will be employed and images of the composites will be provided by optical confocal microscopy. Subsequently, the CO_2_ sensing performance of the composites will be subjected to experimental evaluation.

## Materials and methods

### Materials and instrumentation

Zinc acetate and Copper chloride were purchased from Sigma-Aldrich Company, Inc, USA. Sodium hydroxide and ethanol were purchased from El Nasr Pharmaceutical Chemicals Co., Cairo, Egypt. Phosphoric acid (85%), potassium permanganate (99%), and graphite powder were acquired from Fisher Chemical. Meanwhile, sulfuric acid (96%) was acquired from Scharlau and hydrogen peroxide from PIOCHEM (30%). Deionized (DI) Milli-Q water was used during this experiment. X-ray diffraction (XRD) profiles were scanned at 40 mA and 40 kV of a copper source in the 5°–80° 2-θ range and 0.05° step using a state-of-the-art powder diffractometer, the Bruker D8 Advance (Germany). The crystallinity index was determined using the DIFFRAC.EVA program^25^.

The HighScore suite was employed for analyzing and visualizing the XRD results^26^.The Attenuated Total Reflection Fourier Transform Infrared (ATR-FTIR) spectra were obtained employing an FTIR spectrometer (Vertex 70, Bruker); the spectra are determined within a spectral range of 4000–400 cm^−1^ with a spectral resolution of 4 cm^−1^. A confocal microspectroscopy system which implemented in a WITec alpha 300 R (Germany) confocal Raman microscope capable of high spectral resolution and rapid scanning was utilized to conduct mapping for the detailed surface morphology of the studied samples (GO/ZnO composite and GO/CuO composite). 

### GO preparation

GO was synthesized using the modified version of Hummer’s method. Initially, 3 g of graphite flakes were combined with concentrated sulfuric acid and phosphoric acid mixture in a 9:1 ratio (360 ml sulfuric acid to 40 ml phosphoric acid) while stirring continuously. The mixture was then cooled in an ice bath, and 18 g of potassium permanganate (KMnO_4_) was gradually added. During this process, the color transitioned from black (indicating the presence of graphite) to a dark olive green. To mitigate the exothermic effects, the temperature was maintained between 0 and 5 °C. After stirring the mixture overnight at 40 °C, it was combined with 400 ml of iced deionized water containing 30% hydrogen peroxide (H_2_O_2_). This step resulted in a color change from a buffer violet to light brown. The mixture was then filtered, and the filtrate underwent centrifugation at 10,000 rpm to wash it, discarding the supernatant. Finally, the collected filtrate was dried in an oven at 70 °C for 5 h.

### ZnO preparation

ZnO nanoparticles were synthesized using a precipitation method similar to that described in our earlier research^27,28^, Initially, 0.2 mol of zinc acetate was dissolved in 100 ml of deionized water and stirred vigorously with a magnetic stirrer until fully dissolved. This zinc acetate solution was then heated to 60 °C for approximately 30 min. Meanwhile, a solution of 0.4 mol NaOH was prepared by dissolving it in 50 ml of deionized water. The NaOH solution was gradually added to the zinc acetate solution while maintaining strong stirring for three hours, leading to the formation of white precipitates. The resulting mixture was thoroughly washed with deionized water and filtered. The collected sample was further dried in a dryer at 80 °C for 2.5 h. Finally, the white precipitate was calcined at 500 °C for two hours to ensure the removal of any hydroxyl or carbonyl groups.

### CuO preparation

CuO nanoparticles were synthesized following the procedures outlined in reference^28,29^, initially; 0.2  mol copper chloride was dissolved in 100 ml of deionized water and stirred with a magnetic stirrer. Sodium hydroxide (NaOH) pellets were then added to this solution to achieve a concentration of 0.4  mol. The mixture was heated to approximately 50 °C for four hours while maintaining a pH of eight. Over time, black precipitates began to form at the bottom of the beaker. The resulting particles were collected, centrifuged at 8000 rpm for 10 min, and washed three times with deionized water and once with ethanol. The collected residue was then transferred to an oven and dried at 80 °C for 2 h before being calcined at 500 °C for an additional 2 h to obtain CuO nanoparticles. This process was repeated to produce the necessary quantity of CuO-NPs for subsequent experiments.

### Metal oxides/GO preparation

The PLA substrate was fabricated using a handmade 3D printer to ensure consistent dimensions and thickness. For composite preparation, 200 mg of each pre-synthesized composite material (GO/ZnO, GO/CuO) was mixed with 20 ml of deionized water and sonicated for 20 min. Sonication helped achieve uniform dispersion of the metal oxide and graphene oxide particles in the solution, preventing agglomeration. The resulting solution was drop-cast onto the PLA substrate to create a thin, uniform film. To dry the films, an Nd: YAG laser with a wavelength of 1064 nm, 7 ns pulse duration, 10 Hz pulse repetition rate, and 3.6 W power was used. The laser beam was passed through a quartz concave lens with a focal length of 100 mm, ensuring precise energy delivery. Laser drying was chosen over conventional methods for its ability to deliver rapid and uniform drying while minimizing internal stresses caused by solvent evaporation.

### Sensitivity measurement

The sensitivity as a percentage was calculated using the following equation considering that R_a_ is resistance for air or baseline and R_g_ is resistance for gas^30^. 

$$\:\text{Sensitivity}\left(\text{\%}\right)\:=\:\:\frac{{R}_{a}-{R}_{g}}{{R}_{a}}\:\times\:100$$

**Fig. 1:**
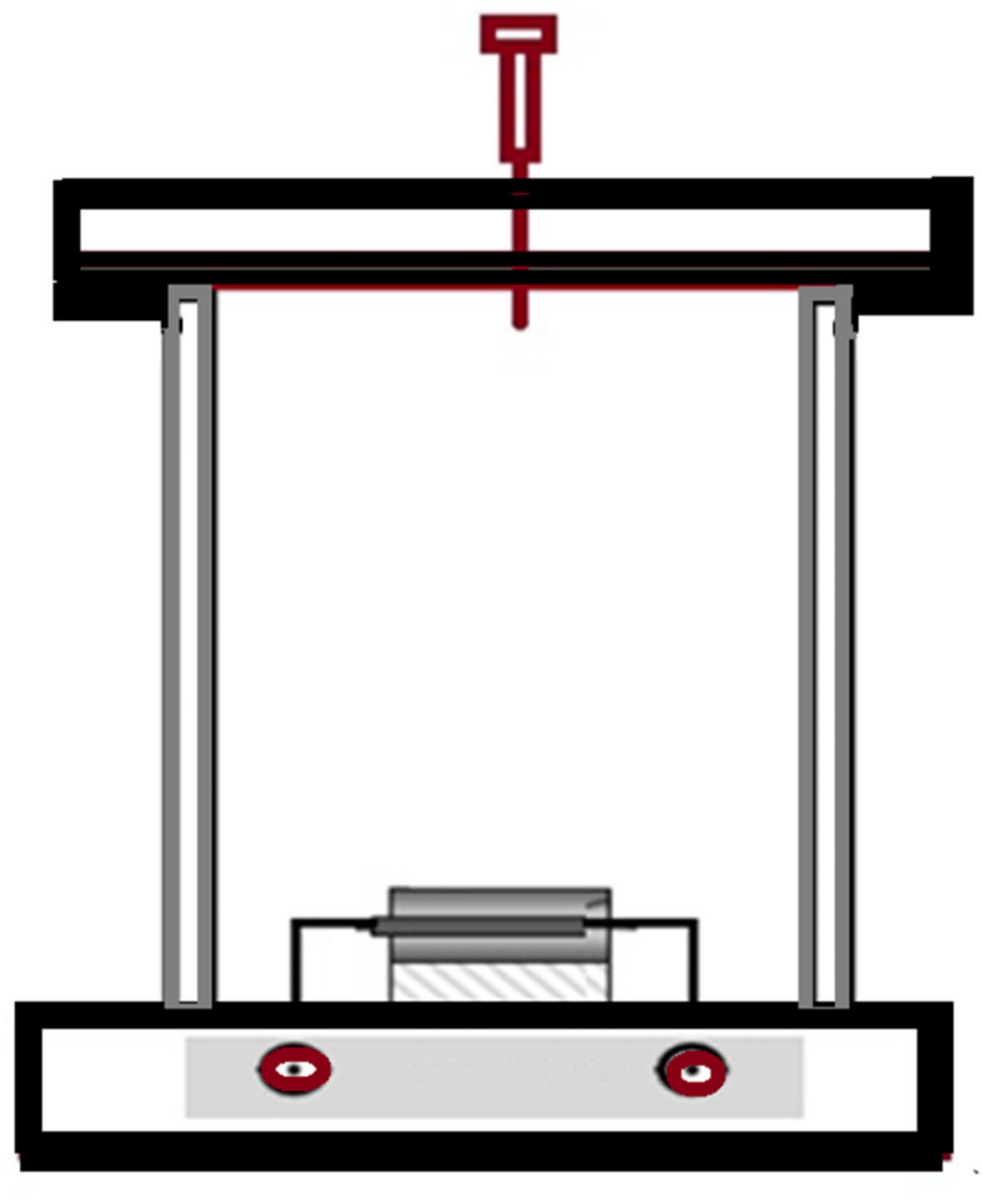
Schematic diagram of the proposed gas sensor chamber which is used for the implementation of gas sensing experiment.

To test the ability of the prepared films to sense gases a chamber is indicated as in Fig.1.

The idea is to inject a known value of gas in a known volume of the chamber, and then the concentration of the studied gas could be calculated. The sensor is placed inside the chamber with electrical contact to connect the sensor with the measured circuit.

### Calculation details

All structures were calculated using the Gaussian 09 program^31^ at the Molecular Modeling and Spectroscopy Laboratory, Centre of Excellence for Advanced Science, NRC, Egypt.

The B3LYP hybrid functional, which is a combination of Becke’s three-parameter exchange functional (B3) and the Lee-Yang-Parr correlation functional (LYP), is a widely utilized approach in computational chemistry. It provides an optimal balance between accuracy and computational efficiency for a diverse range of chemical systems.

So that, each structure underwent optimization with Becke’s three-parameter exchange functional and the Lee-Yang-Parr correlation functional B3LYP^32–34^ alongside the Los Alamos National Laboratory 2 double ζ LANL2DZ basis set. LANL2DZ basis set was developed to address the computational challenges associated with heavy atoms. This hybrid basis set incorporates effective core potentials (ECPs) to replace the core electrons of heavy atoms, which are tightly bound to the nucleus and have minimal influence on chemical bonding. By reducing the number of electrons included in explicit calculations, ECPs significantly lower the computational cost. The double-zeta nature of LANL2DZ ensures that each valence electron is described by two functions, providing a reasonable level of accuracy for molecular properties. The TDM and ∆E values, as well as DOS, were calculated using the same theoretical level.

## Results and discussion

### Building model molecules

**Fig. 2:**
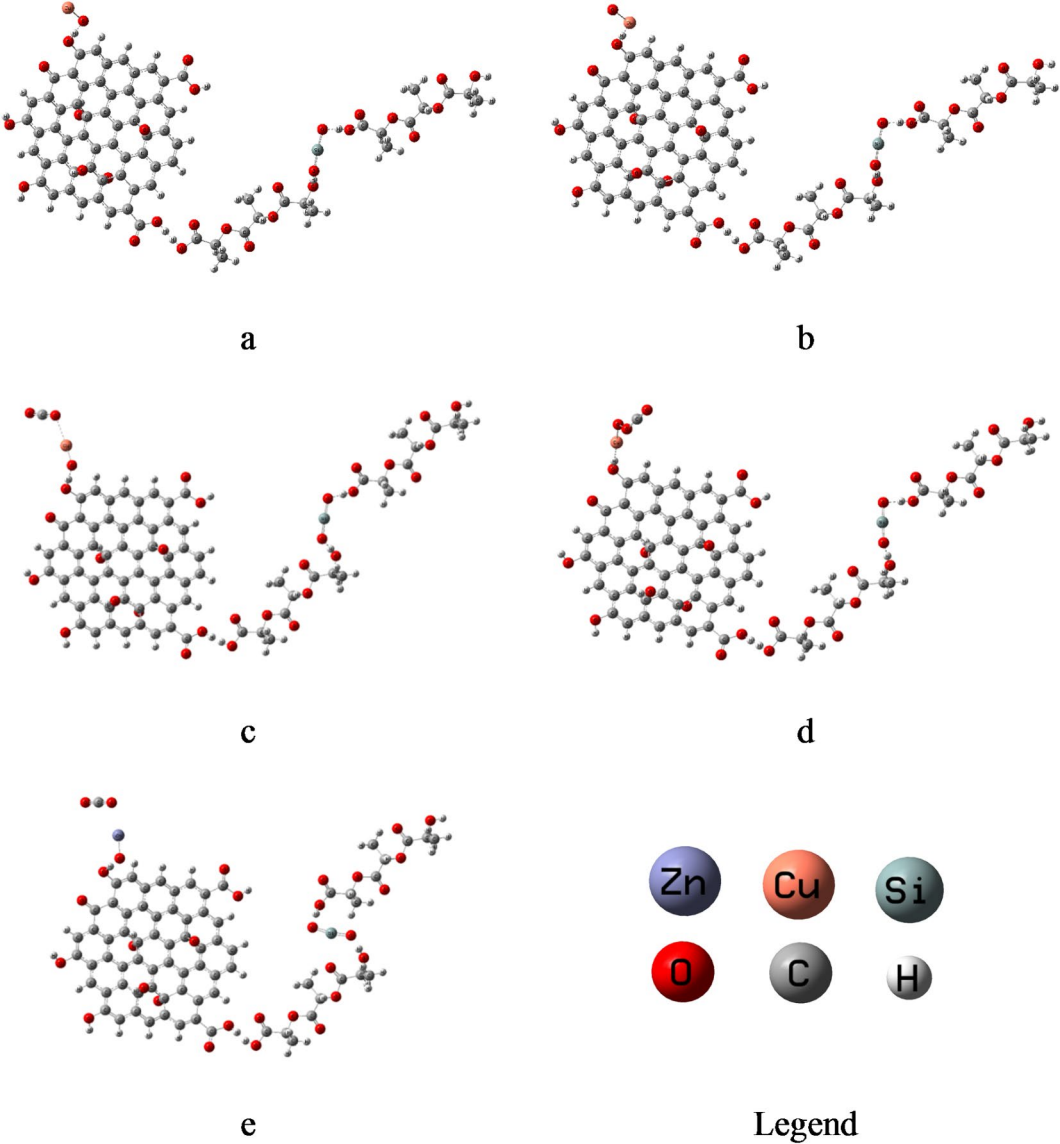
Studied model molecules for (**a**) PLA-SiO_2_/GO/OCu, (**b**) PLA-SiO_2_/GO/CuO, (**c**) PLA-SiO_2_/GO/OCu/CO_2_, (**d**) PLA-SiO_2_/GO/CuO/CO_2_ and (**e**) PLA-SiO_2_/GO/OZn/CO_2_.

Three units of PLA chain modified by SiO_2_ interact weakly with GO from the terminal through OH which is the most active sites GO to form PLA-SiO_2_/GO. This composite was subsequent interacted weakly with CuO and ZnO for CO_2_ sensing applications. Fig. 2 shows the schemes of interactions of PLA-SiO_2_ with GO, ZnO, CuO and CO_2_. Metal oxides interact with PLA-SiO_2_/GO through the OH of GO, which is the most active site. Fig. 2a PLA-SiO_2_/GO is supposed to interact with CuO through the O atom to form PLA-SiO_2_/GO/OCu. Fig. 2b presents the other possibility for interaction of the composite with CuO through the Cu atom forming PLA-SiO_2_/GO/CuO. Fig. 2c presented the interaction between PLA-SiO_2_/GO/OCu and CO_2_ through the Cu atom to form PLA-SiO_2_/GO/OCu/CO_2_. Fig. 2d presents the weak interaction between PLA-SiO_2_/GO/CuO and CO_2_ through the O atom forming PLA-SiO_2_/GO/CuO/CO_2_. The interaction between PLA-SiO_2_/GO and ZnO is supposed to be weak interaction through the O atom of ZnO then it interacts with CO_2_ from Zn atom to form PLA-SiO_2_/GO/OZn/CO_2_ as shown in Fig. 2e. The calculated TDM and ΔE for all studied structures and their interaction with CO_2_ are recorded in Table 1. For PLA-SiO₂/GO/OCu TDM decreases from 13.821 to 11.016 after CO₂ adsorption, while PLA-SiO₂/GO/CuO TDM decreases from 21.335 to 16.290 Debye. This reduction in TDM indicates that the CO₂ adsroption leads to charge redistribution within the composite. The interaction of CO₂ molecules with active sites neutralizes the inherent polarity of these sites, resulting in a lower dipole moment.

For PLA-SiO₂/GO/OCu, ΔE increases slightly from 0.1426 eV to 0.1488 eV, whereas for PLA-SiO₂/GO/CuO, ΔE decreases significantly from 0.3116 eV to 0.1459 eV. The substantial reduction in ΔE for CuO upon CO₂ adsorption highlights a strong interaction between the gas and the composite, which alters the electronic structure by bringing the HOMO and LUMO levels closer, enhancing electronic reactivity. In contrast, the minimal change in ΔE for OCu suggests weaker interactions with CO₂, likely due to differences in the electronic states or surface reactivity between the two materials.

For PLA-SiO₂/GO/OZn/CO₂, energy gap value of 0.2879 eV and TDM 10.608 Debye., suggest that the incorporation of SiO₂ into PLA improves the structural and electronic properties of the composite compared to PLA/GO/OZn from our previous study^21^, Similary, the TDM and ΔE values of PLA-SiO₂/GO/CuO (16.290 Debye and 0.1459 eV, respectively) are superior to those of PLA/GO/Cu₂O/CO₂ reported in our earlier work^21^ (10.650 Debye and 1.038 eV, respectively). This demonstrates that the addition of SiO₂ to PLA and the replacement of Cu₂O with CuO in the composite positively enhance its structural and electronic characteristics.

**Table 1:** B3LYP/LANL2DZ calculated TDM as Debye and ΔE as eV for the studied model molecules.

Structure	TDM (Debye)	ΔE (eV)
PLA-SiO_2_/GO/OCu	13.821	0.1426
PLA-SiO_2_/GO/CuO	21.335	0.3116
PLA-SiO_2_/GO/OCu/CO_2_	11.016	0.1488
PLA-SiO_2_/GO/CuO/CO_2_	16.290	0.1459
PLA-SiO_2_/GO/OZn/CO_2_	10.608	0.2879

### Mapping molecular electrostatic potential MESP

**Fig. 3:**
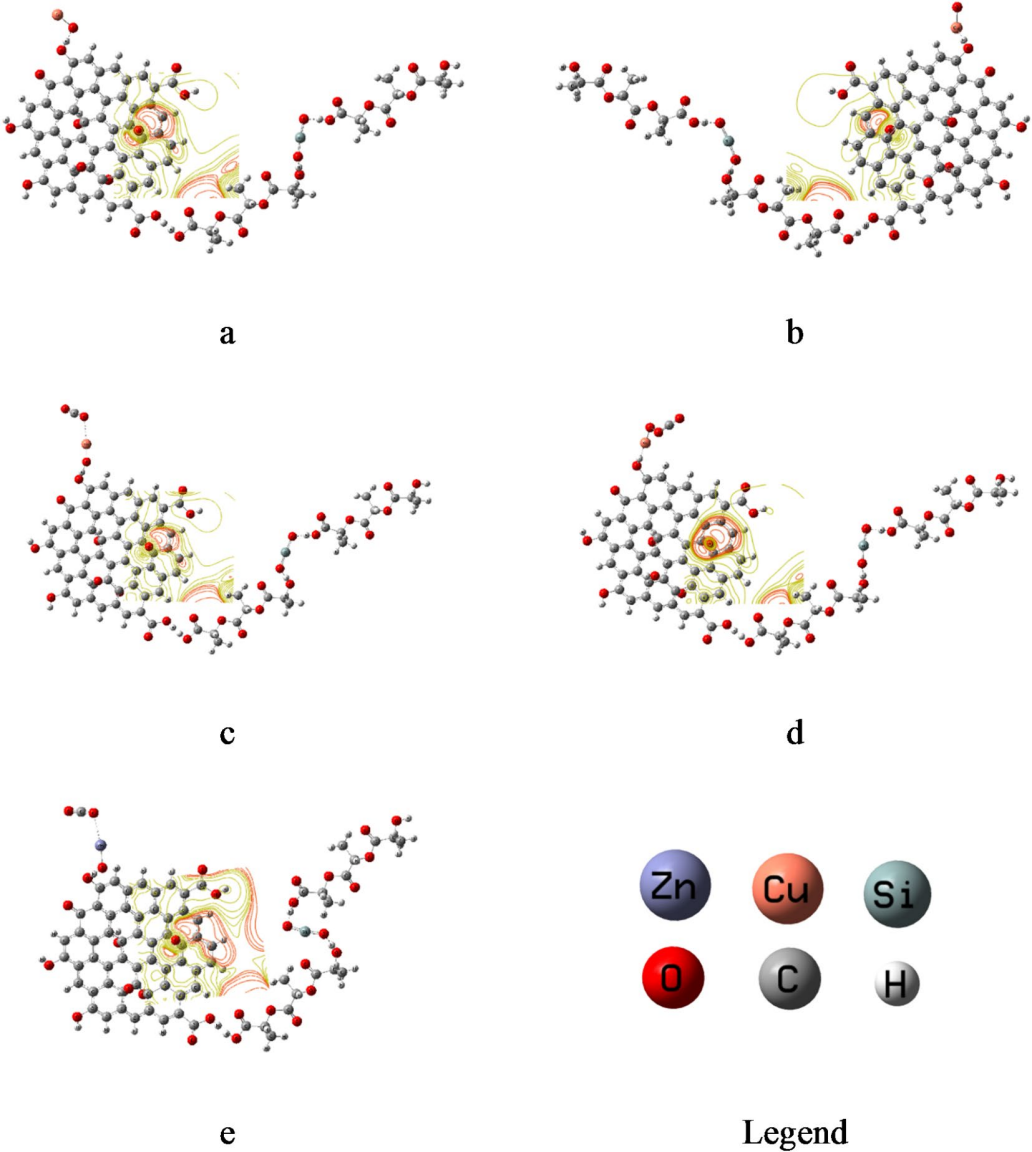
B3LYP/LANL2DZ mapped molecular electrostatic potential MESP for the studied model molecules whereas (**a**) PLA-SiO_2_/GO/OCu, (**b**) PLA-SiO_2_/GO/CuO, (**c**) PLA-SiO_2_/GO/OCu/CO_2_, (**d**) PLA-SiO_2_/GO/CuO/CO_2_ and (**e**) PLA-SiO_2_/GO/OZn/CO_2_.

Fig. 3 presents the B3LYP/LANL2DZ mapped molecular electrostatic potential MESP for the studied model molecules. The composite PLA-SiO₂/GO interacted with CuO displayed a reactive potential around oxygen atom at the edge of GO, manifesting in the red color range. This means that the surface edge of GO becoming more reactive as it is interacted with PLA-SiO₂ and CuO even the interaction from O atom or Cu atom of CuO (Fig. 3a, b). Following the interaction PLA-SiO₂/GO/CuO with CO₂ gas, the composites retained a reactive surface, indicating the potential for further reactions as shown in Fig. 3c, d. The composite PLA-SiO_2_/GO/CuO exhibiting a stronger MESP and higher reactivity towards CO_2_ gas compared to the composite PLA-SiO_2_/GO/OCu. MESP was also calculated for PLA-SiO₂/GO interacted with ZnO from O atom and CO_2_ as shown in Fig. 3e. From the figure, MESP of PLA-SiO_2_/GO/OZn composite still has reactivity towards as CO_2_ but is lower than in the case of PLA-SiO_2_/GO/CuO composite. MESP results confirm the obtained results of TDM and band gap energy ΔE.

### Density of states

**Fig. 4:**
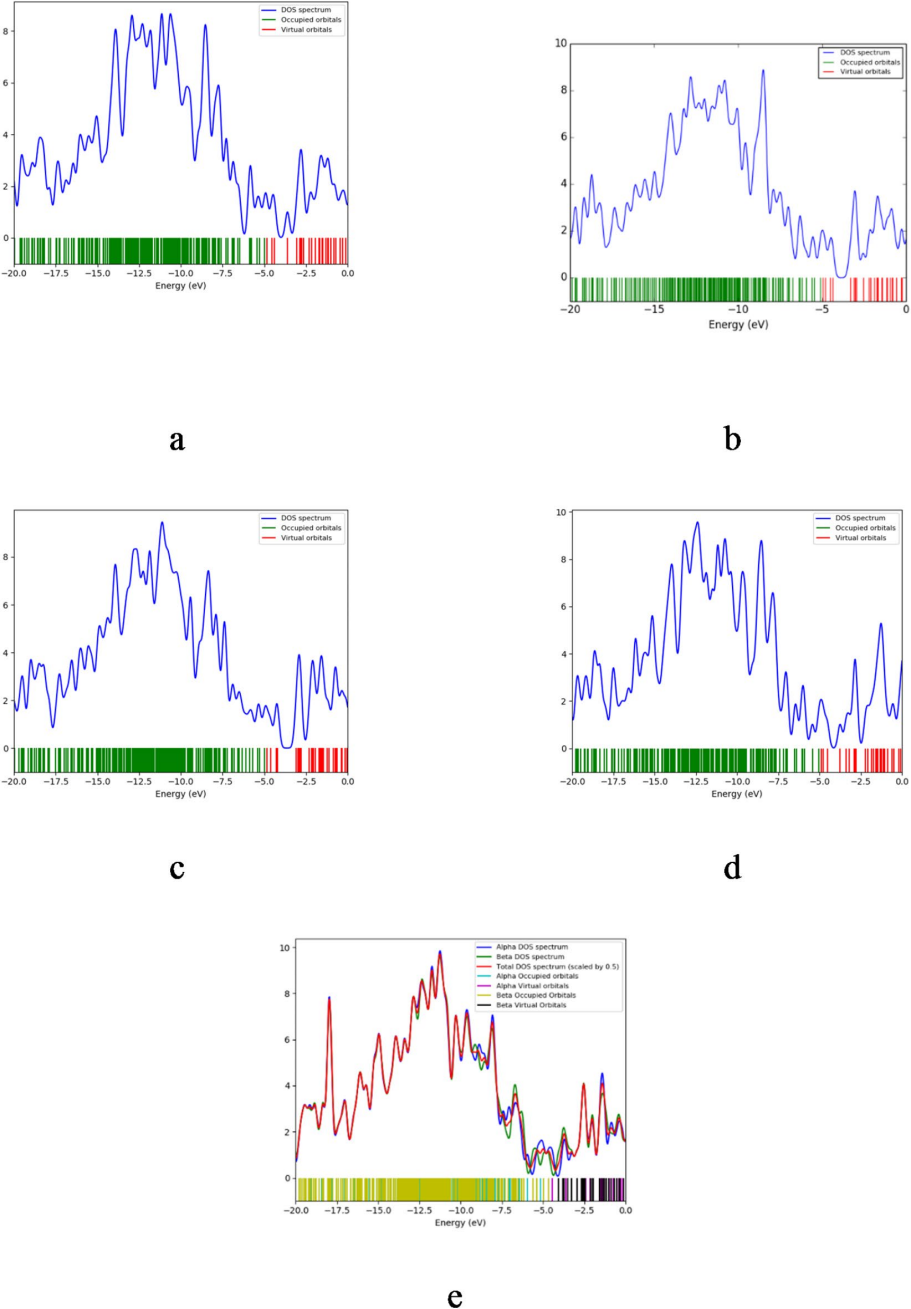
DOS plots of model molecules for the structures (**a**) PLA-SiO_2_/GO/OCu, (**b**) PLA-SiO_2_/GO/CuO, (**c**) PLA-SiO_2_/GO/OCU/CO_2_, (**d**) PLA-SiO_2_/GO/CuO/CO_2_ and (**e**) PLA-SiO_2_/GO/OZn/CO_2_.

The presence of the studied structures on metals makes the DOS an important parameter for studying these composites. It is defined as the distribution of all obtainable quantum states per unit energy of the molecule. In Fig. 4, the plots of DOS spectrum demonstrates a distinct separation between occupied (green) and virtual (red) states, emphasizing the electronic transitions within the composite materials. Furthermore, a minor band gap exists between the valence and conduction bands. The concentration of states in the vicinity of the Fermi level (approximately 0 eV) is of paramount importance in facilitating interactions between the material and CO₂ molecules. A higher density of states near the Fermi level may indicate regions where electron transfer is more probable, thereby enhancing the material’s sensitivity to gas molecules. The e- PLA-SiO₂/GO/OZn/CO₂ composite exhibits a band gap of 0.288 eV, confirming its semiconducting nature. The DOS plot demonstrates a high density of states in the vicinity of the Fermi level (0 eV), indicating a substantial potential for electron transfer during CO₂ adsorption. The balanced alpha and beta DOS spectra imply weak spin polarization, indicating a uniform electronic distribution, which is advantageous for stable charge transport in gas sensing applications. These electronic properties indicate that the PLA-SiO_2_/GO/OZn composite is well-suited for CO₂ sensing due to its favorable reactivity and charge transfer kinetics.

### X-ray diffraction characterization

The crystallinity index was determined using the DIFFRAC.EVA program^25^. The HighScore suite was employed for analyzing and visualizing the XRD results^26^.

**Fig. 5:**
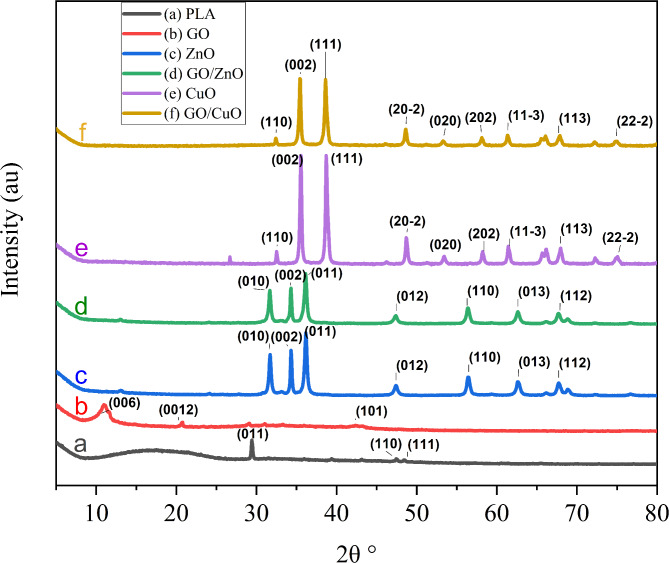
XRD graph for the composites (a) PLA, (b) GO, (c) CuO, (d) GO/CuO, (e) ZnO and (f) GO/ZnO.

The X-ray diffraction (XRD) pattern results in Fig. 5 demonstrated the presence of polylactic acid (PLA), which exhibited a broad diffraction peak at 2θ = 14.044° and a characteristic peak.

at 2θ = 29.433°. The broad peak verifies the amorphous nature of PLA, while the sharp peaks indicate the presence of silicon oxide (SiO_2_), which is consistent with the PDF card number 98-015-5252.

X-ray diffraction (XRD) of graphene oxide (GO) exhibits a broad peak around 2θ = 10.944°, larger than that of graphite. This is due to the presence of oxygen-containing functional groups on the GO, which match the well-known XRD peak of GO. Furthermore, broad peaks are observed between 27° and 44°. These peaks can be interpreted in terms of the short-range order in the stacked graphene sheets. The presence of copper and zinc oxides was confirmed by observing distinctive peaks in the X-ray diffraction patterns and comparing them with the PDF reference cards Nos. 98-062-8616 and 98-006-5121, respectively.

Table 2 presents the results of the crystallinity index and crystallite size. Due to GO’s amorphous nature, the addition of GO to ZnO and CuO decreases crystallinity and crystallite size.

These analyses confirm the formation of GO/CuO and GO/ZnO composites, which exhibit enhanced structural and chemical properties due to the interaction between GO, CuO, and ZnO.

**Table 2:** Crystallinity index of the samples PLA, GO, CuO, GO/CuO, ZnO and GO/ZnO respectively.

Sample	Crystallinity index %	Crystallite Size (nm)
PLA	47	60
GO	47	48
CuO	86	774
GO/CuO	77	104
ZnO	80	135
GO/ZnO	78	28

### FTIR characterization

**Fig. 6:**
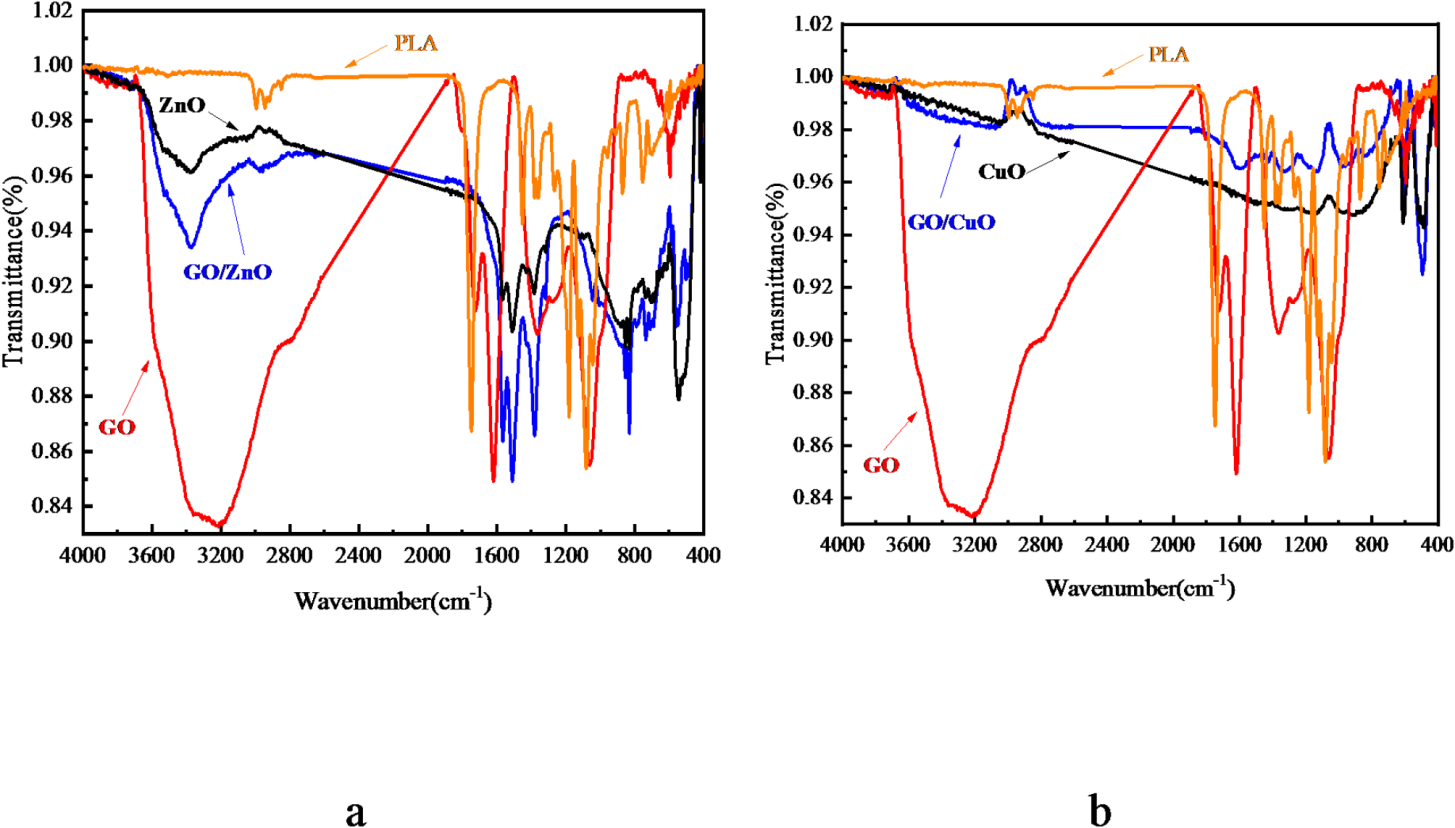
FTIR spectrum for the studied samples whereas, (**a**) PLA, GO, ZnO, and GO/ZnO composite, (**b**) PLA, GO, CuO, and GO/CuO composite.

In Fig. 6a, the FTIR spectrum analysis of the GO/ZnO composite reveals several significant findings concerning the interactions and structural characteristics of the materials.

A spectral shift from 703.72 cm^−1^ to 735.57 cm^−1^ suggests significant interactions between ZnO and graphene oxide (GO), likely indicating the formation of new chemical bonds or changes in the electronic environment within the composite material^35^.

At 854.85 cm^−1^, the consistent band in both ZnO and GO/ZnO spectra indicates the stability of the Zn-O bond structure, implying that the incorporation of GO does not disrupt the integrity of the Zn-O bonding network^35^.

At 1383.25 cm^−1^, the C-H bending vibrations indicate the presence of organic components in both ZnO and GO/ZnO, potentially residual compounds from synthesis or intentional doping with organic molecules^36^.

The peak at 565.16 cm^−1^ corresponds to C-H stretching or bending vibrations typically associated with aromatic groups in graphene oxide, confirming the successful incorporation of GO into the composite material.

The presence of a band at 1081.39 cm^−1^ due to Si-O stretching in PLA suggests SiO₂ as an impurity^37^. Slight shifts in PLA bands (1043.78, 1081.39, 1127.53, and 1180.69 cm^−1^) indicate possible interactions between PLA and SiO₂. The broadening of some bands in the composite spectrum may indicate overlapping or broadening of the Si-O and PLA bands due to interactions or formation of new chemical bonds^36^.

The presence of a band at 1619.48 cm^−1^ in the GO spectrum indicates C = C stretching vibrations characteristic of the graphene structure within GO, highlighting the retention of graphene-like properties in the oxide form. In both ZnO and GO/ZnO spectra, bands at 1565.68 and 1510.61 cm^−1^ could suggest carbon-based impurities, while the identical 1510.61 cm^−1^ band in both materials points to interactions between ZnO and GO, affecting the composite’s C = C groups^35^.

The observed O-H stretching vibrations at 3374.56 cm^−1^ in ZnO are most likely caused by water adsorption on the sample surface. In GO/ZnO, this band indicates the presence of hydroxyl groups as well, suggesting strong interactions or bonding between ZnO and GO.

Furthermore, the band at 3216.21 cm^−1^ is characteristic of hydroxyl groups specifically in graphene oxide (GO), confirming the presence of these functional groups within the composite material^38^, while in Fig. 6b, the FTIR spectral analysis of CuO and GO/CuO composites provides valuable insights into their structural and electronic interactions. The characteristic band of the Cu-O bond at 611.29 cm^−1^ in both CuO and GO/CuO indicates the presence of copper oxide. This band reflected specific vibrational modes that signify bond stiffness and atomic mass. This consistent band suggests that the fundamental lattice structure involving Cu-O bonds remains unchanged in the composite^39^.

The C-O bond observed at 1138.87 cm^−1^ in both CuO and GO/CuO samples indicates the presence of similar carbon-oxygen functional groups^40^, likely due to interactions between carbon in GO and oxygen in CuO. This vibrational frequency reflects the strength of the C-O bond and the electronic environment influenced by copper oxide. In contrast, the C-O bond at 1367.43 cm^−1^ in pure GO indicates a different type of C-O bonding environment compared to GO/CuO^38^, implying distinct vibrational modes due to the absence of copper oxide interactions. This suggests that the electronic environment around C-O bonds in pure GO is primarily influenced by its intrinsic properties.

The shift of the C=C bond band from 1619.48 cm^−1^ in GO to 1594.30 cm^−1^ in GO/CuO indicates significant interactions between CuO and GO, resulting in alterations to the electronic environment. This shift signifies changes in the π-electron distribution of the double bond, indicating potential chemical bonding or charge transfer effects between GO and CuO. Such alterations in bond strength and electronic distribution highlight the complex interplay induced by the incorporation of CuO into GO.

The presence of O-H stretching vibrations at 3216.21 cm^−1^ in GO indicates the presence of hydroxyl groups, typically reflecting their bond strength and hydrogen bonding environment. In pure GO, these groups are either free or weakly interacting within the matrix^40^. The absence of this band in GO/CuO suggests altered hydroxyl interactions in the composite, potentially involving new bonds or deprotonation. Conversely, the O-H stretching vibration at 3706.77 cm^−1^ is present in both CuO and GO/CuO, indicating that the hydroxyl environment in CuO remains stable in the composite. This contrast suggests that while GO’s hydroxyl groups are modified, those in CuO retain their original bonding characteristics and electronic environment.

The FTIR spectra of both GO/ZnO and GO/CuO composites underscore structural and electronic interactions due to GO incorporation. In the GO/ZnO composite, distinct spectral shifts and modifications compared to pure ZnO indicate substantial interactions between ZnO and GO, potentially involving new chemical bonds or alterations in the electronic environment. For instance, the shift of the Zn-O bond band from 703.72 cm^−1^ to 735.57 cm^−1^ suggests changes in the electronic environment or the formation of chemical bonds between ZnO and GO. The broadening of Si-O and PLA bands in the GO/ZnO composite, such as at 1043.78 and 1081.39 cm^−1^, further indicates physical interactions like van der Waals forces or dipole-dipole interactions, rather than the formation of new chemical bonds. These complementary physical and chemical interactions stabilize the composite matrix and enhance its structural properties, making it suitable for applications in electronics, sensors, and catalysis.

Similarly, the FTIR analysis of the GO/CuO composite reveals notable shifts in vibrational frequencies, indicating significant changes in bond strengths and electronic environments upon integrating CuO with GO. The shift of the C = C bond band from 1619.48 cm^−1^ in GO to 1594.30 cm^−1^ in GO/CuO highlights strong interactions, such as charge transfer effects or chemical bonding. Furthermore, the disappearance of the O-H stretching vibration at 3216.21 cm^−1^ in GO/CuO indicates modified hydroxyl interactions, likely involving new bonds or deprotonation. Meanwhile, the stability of the Cu-O bond at 611.29 cm^−1^ underscores that CuO retains its fundamental lattice structure, supporting selective chemical interactions without disrupting its integrity.

The FTIR analysis demonstrates how GO incorporation influences both structural and electronic properties in these composites through a combination of physical and chemical interactions. Spectral shifts, such as those in Zn-O and C = C bonds, suggest localized chemical bonding or charge transfer effects, while vibrational broadening reflects physical interactions that stabilize the matrix.

### Confocal microscopy

**Fig. 7:**
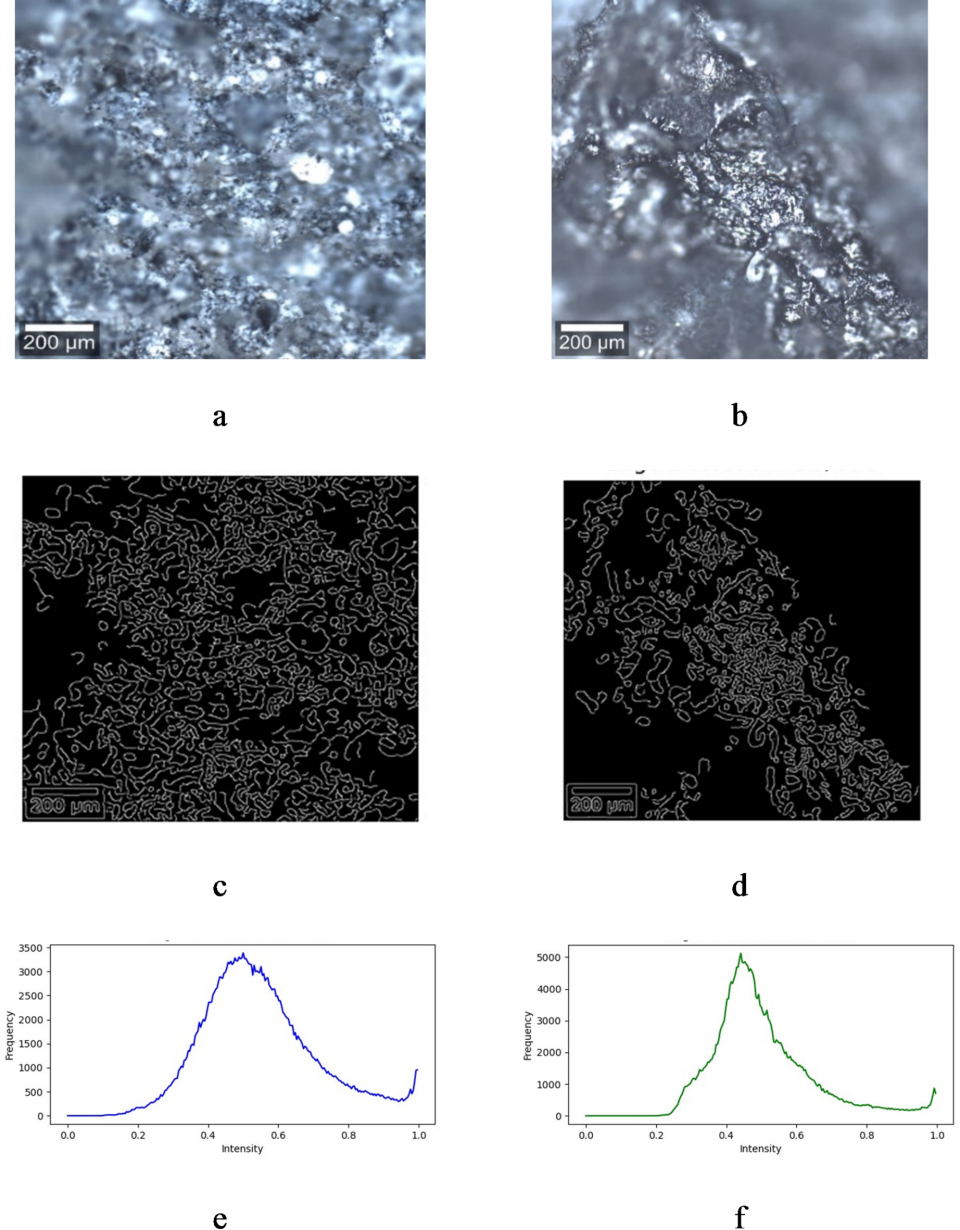
Confocal microscopy of the sample whereas high-resolution original images, edge detection and intensity shown respectively in (**a**,**c**,**e**) as GO/ZnO and (**b**,**d**,**f**) as GO/CuO.

Fig. 7 shows the detailed surface morphology of each sample, with Fig. 7a displaying the GO/ZnO composite and Fig. 7b displaying the GO/CuO composite. The brightness histogram analysis of the confocal microscopy images revealed notable differences in the pixel intensity distribution for the GO/ZnO and GO/CuO composites. GO/ZnO and GO/CuO composites exhibited sharp peaks at an intensity level of 0.5 and 0.44 respectively; corresponding to mid-gray brightness, indicating the presence of uniformly distributed surface features. However, the frequency of pixels at this intensity was significantly higher for GO/CuO (5122 pixels) compared to GO/ZnO (3385 pixels). This distinction suggests that the surface morphology of the GO/CuO composite is more homogeneous, likely characterized by finer or more evenly dispersed features. In contrast, the lower frequency observed for GO/ZnO implies a greater heterogeneity, potentially due to the presence of larger aggregates or more uneven distribution of ZnO particles on the GO matrix. These differences in surface texture and brightness uniformity reflect variations in the interaction between the GO matrix and the metal oxides, influenced by particle size, dispersion, and the bonding nature of the GO-metal oxide phases. Such findings are important to understand the structural and functional properties of these composites. The higher uniformity of GO/CuO aligns with its superior gas sensing performance, offering enhanced sensitivity and reproducibility due to its consistent surface properties. Conversely, the heterogeneous surface of GO/ZnO may contribute to unique gas-sensing mechanisms by providing larger reactive surface areas, which could benefit selectivity or adsorption-based sensing.

### Sensing for CO_2_

The sensing experiment was designed to evaluate the effectiveness of thin-film sensors in detecting gaseous substances, focusing on CO_2_. It involved preparing composite-based sensing films, integrating them onto a PLA substrate, and testing them in a controlled environment. This approach allowed for the assessment of the films’ sensitivity, response time, and potential for practical applications in gas detection.

The experiment had two main stages: preparing the sensing films and evaluating their performance. During preparation, various composite materials were mixed, sonicated, and dried using a laser technique to form uniform thin films on a PLA substrate. These films were then placed in a gas chamber equipped with electrical contacts to measure changes in their resistance when exposed to CO_2_ gas.

The results provided valuable insights into the films’ ability to detect gas presence and concentration changes. The rapid and consistent decrease in resistance upon exposure to CO_2_ indicated a high sensitivity, showcasing their potential as effective gas sensors. This experiment demonstrated the feasibility of using composite materials for gas sensing and laid the groundwork for further development and optimization of such sensors for various applications.

When CO_2_ gas is injected into the gas chamber indicated in Fig. 1, the resistance of the element rapidly decreases and quickly reaches a constant value. It is discovered that the resistance of the all-sensing film decreases in the presence of CO_2_ gas.

**Fig. 8:**
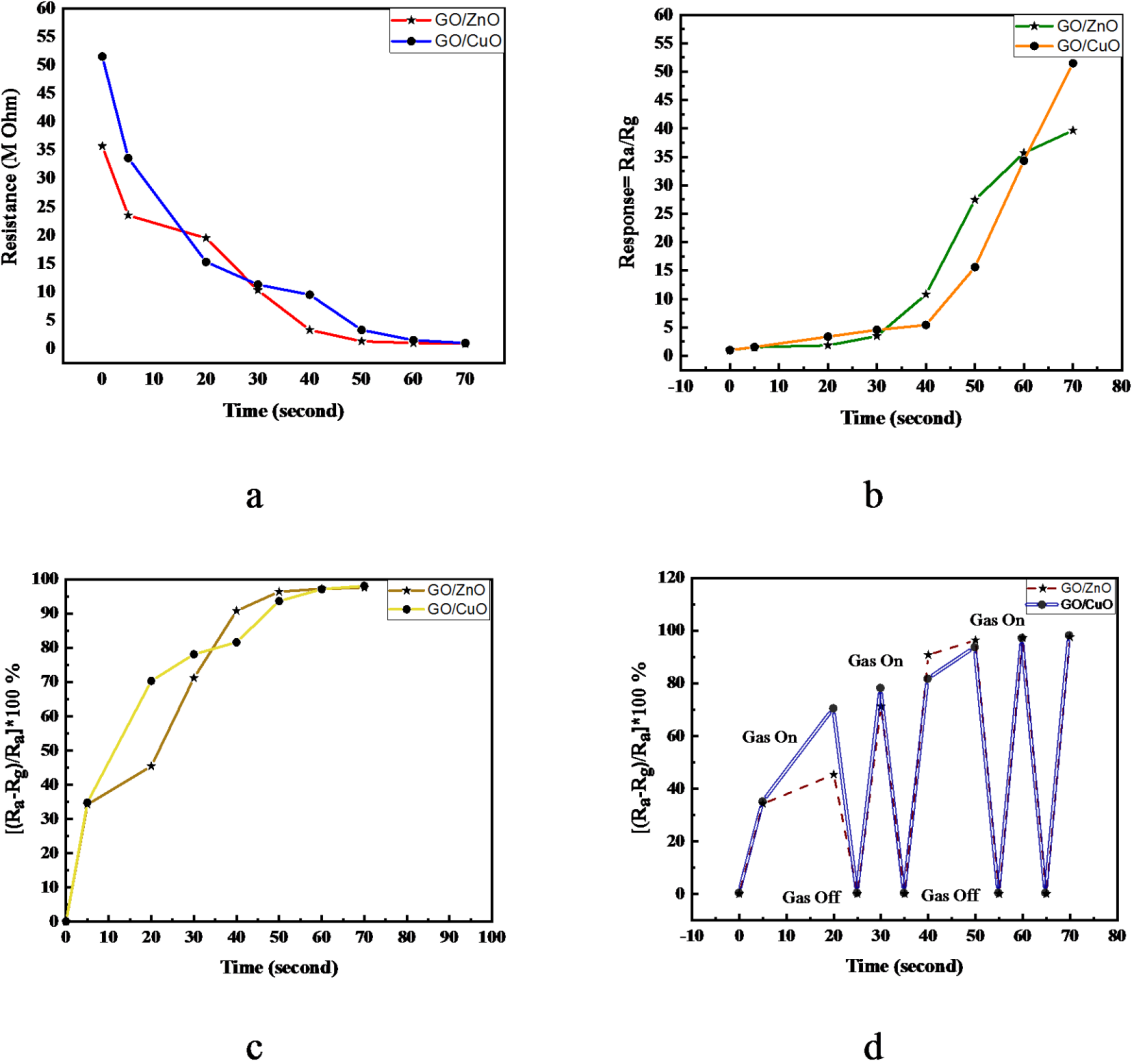
Sensing experiment with CO_2_ Gas for the studied composites GO/ZnO and GO/CuO whereas, (**a**) Resistivity, (**b**) Response, (**c**) Sensitivity with continuous gas, (**d**) Sensitivity with discrete gas quantity.

Both GO/ZnO and GO/CuO composites demonstrate a significant decrease in resistance upon CO₂ exposure Fig. 8a, indicating their sensitivity to gas adsorption. GO/ZnO, with an initial resistance of 35.7 Ω, exhibits a sharp decline stabilizing near zero within 70 s. GO/CuO, starting at a higher resistance (51.5 Ω), also stabilizes near zero with a steeper decline. This suggests efficient electron transfer and adsorption in both composites, with GO/CuO potentially demonstrating a stronger response due to CuO’s inherent electronic and catalytic properties. In Fig. 8b illustrates the dynamic interaction of both composites with CO₂ molecules, as evidenced by the progressive increase in response curves over time. Notably, GO/CuO exhibits a higher peak response compared to GO/ZnO, particularly between 60 and 70 s, suggesting a more robust CO₂ interaction. In Fig. 8c both composites show rapid increase in sensitivity with continuous exposure of the gas. Regarding Fig. 8d, both sensors display a positive response to CO₂, characterized by increased sensitivity upon gas introduction and subsequent decrease upon its removal. This confirms the reversibility and stability of the sensing mechanisms in both composites. Furthermore, a strong correlation between sensitivity and response is observed, indicating that a more sensitive sensor generates a stronger signal in the presence of CO_2_.

**Fig. 9:**
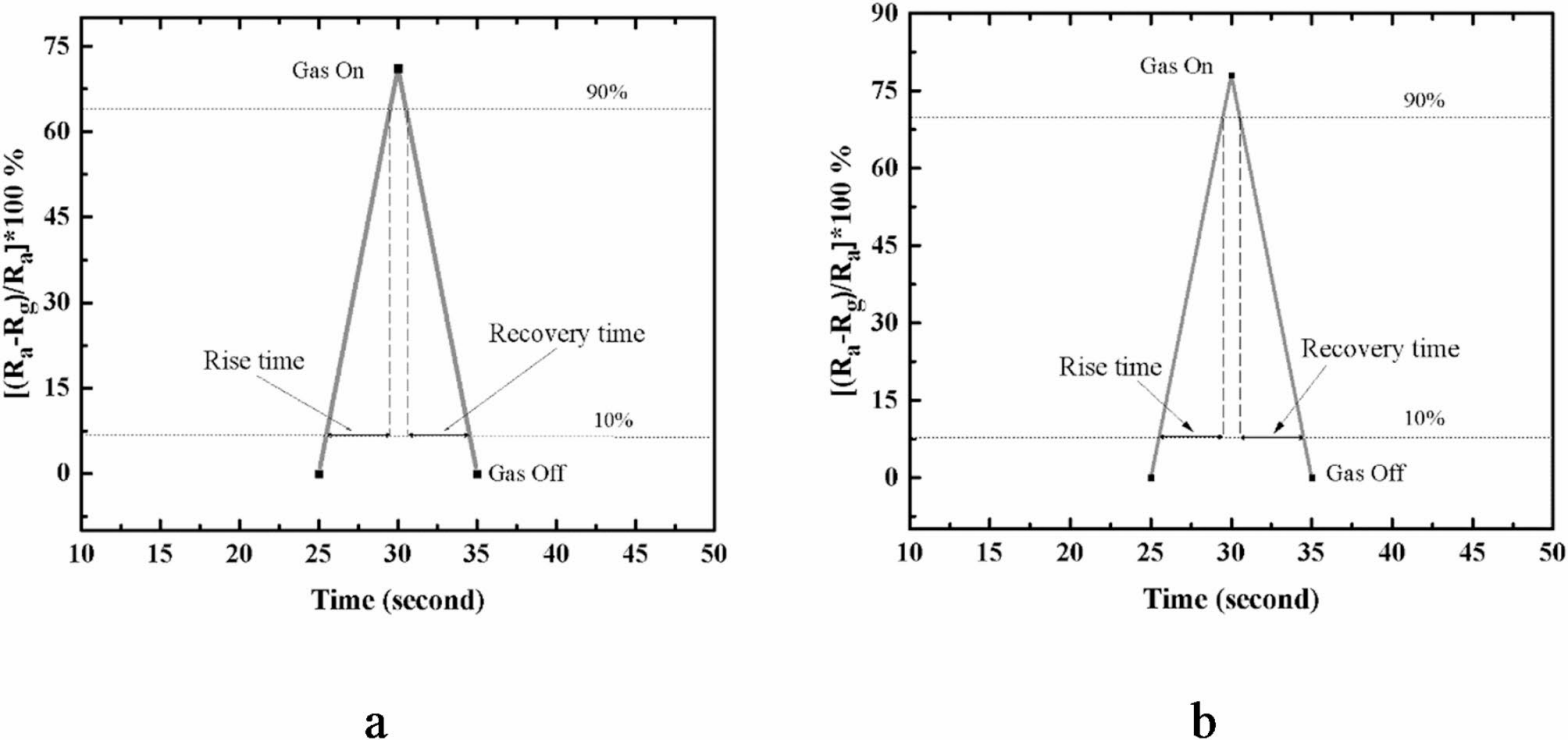
Recovery time and rise time for the studied composites whereas, (**a**) GO/ZnO and (**b**) GO/CuO.

### Recovery time measurement

Fig. 9 demonstrates the dynamic sensitivity and recovery behavior of the GO/ZnO and GO/CuO sensor in the presence and absence of the target gas. The recovery time is defined as the interval required for the sensor signal to return to 90% of its baseline following the removal of the gas, whereas the rise time refers to the time taken to reach 90% of the peak response upon gas exposure^41^. During the recovery phase, it is likely that some gas molecules remain adsorbed on the surface of the composites metal oxides, causing a slight delay in achieving complete baseline stabilization. Consequently, the recovery time is measured from the 90% baseline threshold to provide more representative depiction of the sensor sensitivity^42^. The GO/ZnO and GO/CuO sensors exhibits a consistent recovery time of approximately 4 s across all measurement cycles, suggesting reliable and efficient desorption of gas molecules after each exposure. In contrast, the rise time shows variability, although it remains rapid overall, reflecting the sensor’s capacity for prompt response. The rapid and stable recovery time is indicative of the sensor’s stability and reproducibility, which are critical for practical applications. The consistency of the recovery time across multiple cycles highlights the sensor’s operational stability and durability, confirming its potential for reliable performance in real-time gas sensing applications.

## Conclusion

In this study, the integration of graphene oxide (GO) with ZnO and CuO into polylactic acid (PLA) matrices successfully produced nanocomposites with enhanced structural integrity and improved gas sensing properties. X-ray diffraction (XRD) analysis confirmed the formation of well-defined crystalline phases in both GO/ZnO and GO/CuO composites, which benefited from reduced impurity levels, leading to improved structural stability. Additionally, Fourier-transform infrared (FTIR) spectroscopy indicated some modifications in chemical bonding and the electronic environment of the composites, which are critical for optimizing gas sensing performance. Confocal microscopy revealed a more uniform distribution of CuO within the GO matrix compared to ZnO. Theoretical calculations highlighted the superior adsorption energy and electronic properties of the PLA-SiO₂/GO/CuO composite, suggesting enhanced interaction with CO₂ molecules. Experimental gas sensing results demonstrated that both PLA-SiO₂/GO/ZnO and PLA-SiO₂/GO/CuO composites exhibited dynamic and time-dependent changes in resistance and sensitivity upon exposure to CO₂, with the PLA-SiO₂/GO/CuO sensor demonstrating higher sensitivity and faster response. These findings underscore the potential of these PLA-based nanocomposites as reliable real-time gas sensors for environmental monitoring and industrial safety applications. The composites exhibited rapid rise and recovery times with consistent recovery across cycles, indicating strong sensor stability and suitability for applications requiring swift and repeatable detection cycles.

## Data Availability

The data that support the findings of this study are available from the corresponding author upon reasonable request.
